# Catheterization and embolization of a replaced left hepatic artery via the right gastric artery through the anastomosis: a case report

**DOI:** 10.1186/1752-1947-5-346

**Published:** 2011-08-03

**Authors:** Masaya Miyazaki, Kei Shibuya, Yoshito Tsushima, Keigo Endo

**Affiliations:** 1 Department of Diagnostic and Interventional Radiology, Gunma University Graduate School of Medicine, 3-39-15 Showa-machi, Maebashi, Gunma, Japan

## Abstract

**Introduction:**

Conversion of multiple hepatic arteries into a single vascular supply is a very important technique for repeat hepatic arterial infusion chemotherapy using an implanted port catheter system. Catheterization of a replaced left hepatic artery arising from a left gastric artery using a percutaneous catheter technique is sometimes difficult, despite the recent development of advanced interventional techniques.

**Case presentation:**

We present a case of a 70-year-old Japanese man with multiple hepatocellular carcinomas in whom the replaced left hepatic artery arising from the left gastric artery needed to be embolized. After several failed procedures, the replaced left hepatic artery was successfully catheterized and embolized with a microcatheter and microcoils via the right gastric artery through the anastomosis.

**Conclusion:**

A replaced left hepatic artery arising from a left gastric artery can be catheterized via a right gastric artery by using the appropriate microcatheter and microguidewires, and multiple hepatic arteries can be converted into a single supply.

## Introduction

Conversion of multiple hepatic arteries into a single vascular supply is a very important technique for repeat hepatic arterial infusion chemotherapy using an implanted port catheter system [1-4]. In cases in which a replaced left hepatic artery (LHA) arising from a left gastric artery (LGA) is present, the replaced LHA should be embolized at the proximal portion to convert multiple vascular supplies into a single supply. However, catheterization of an LGA using a percutaneous catheter technique is sometimes difficult, despite recently developed advanced interventional techniques. We report an unusual case of a patient in whom the replaced LHA was catheterized and embolized with a microcatheter through the anastomosis from the right gastric artery (RGA) to the LGA.

## Case presentation

**Figure 1:**
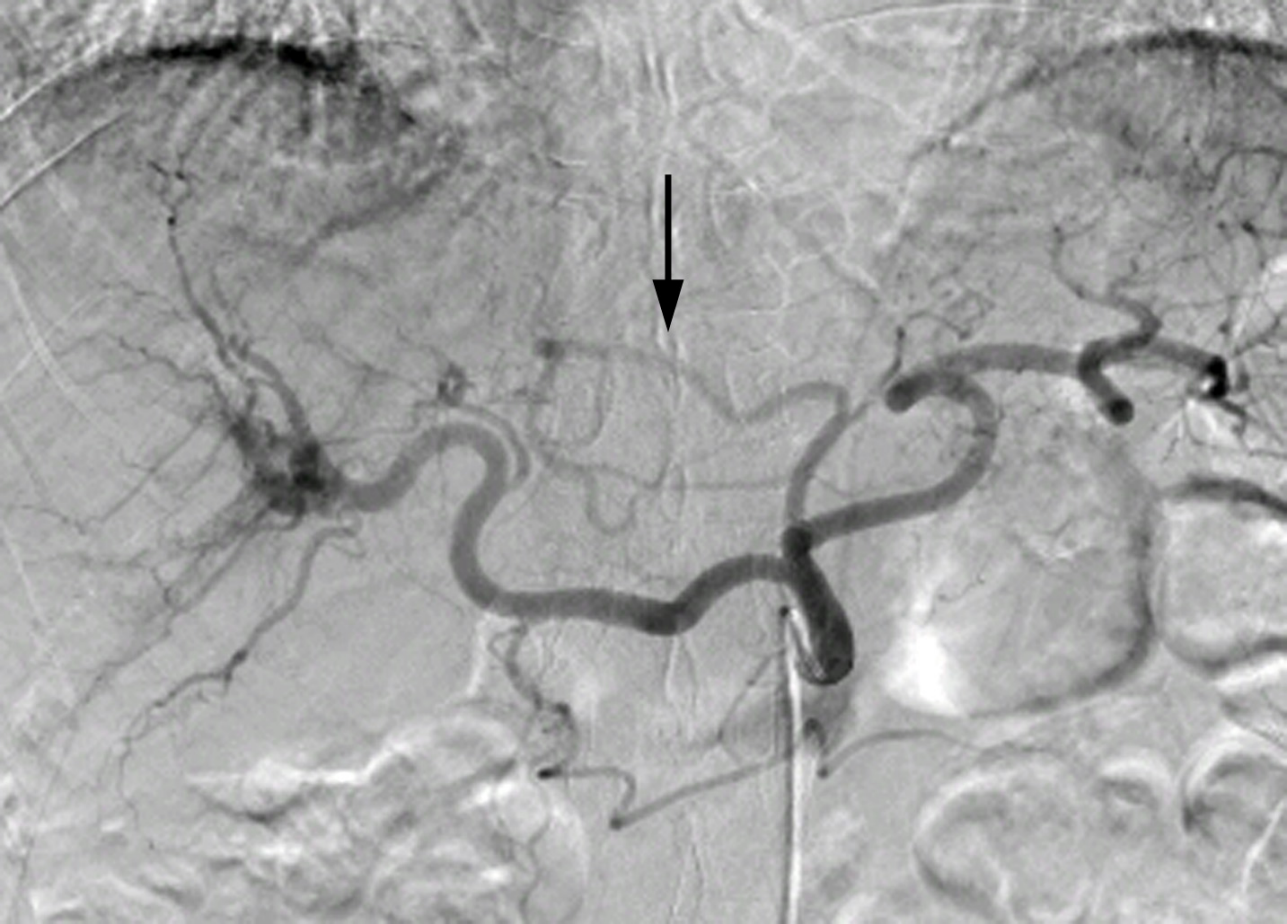
**Celiac angiogram showing the replaced left hepatic artery (arrow) arising from the left gastric artery**.

Our patient was a 70-year-old Japanese man with multiple hepatocellular carcinomas who required repeat multiple transarterial chemoembolization and radiofrequency ablation treatments because of recurrences. Repeat hepatic arterial infusion chemotherapy using an implanted port-catheter system had been planned for the patient in another institution. Since the replaced LHA arose from the LGA (Figure 1), arterial redistribution by means of embolizing the replaced LHA had been attempted. However, despite three procedures, the LGA could not be selected using the catheter, and the replaced LHA could not be catheterized and embolized. Therefore, the patient was transferred to our institution, and arterial redistribution and creation of the port-catheter system were planned.

**Figure 2:**
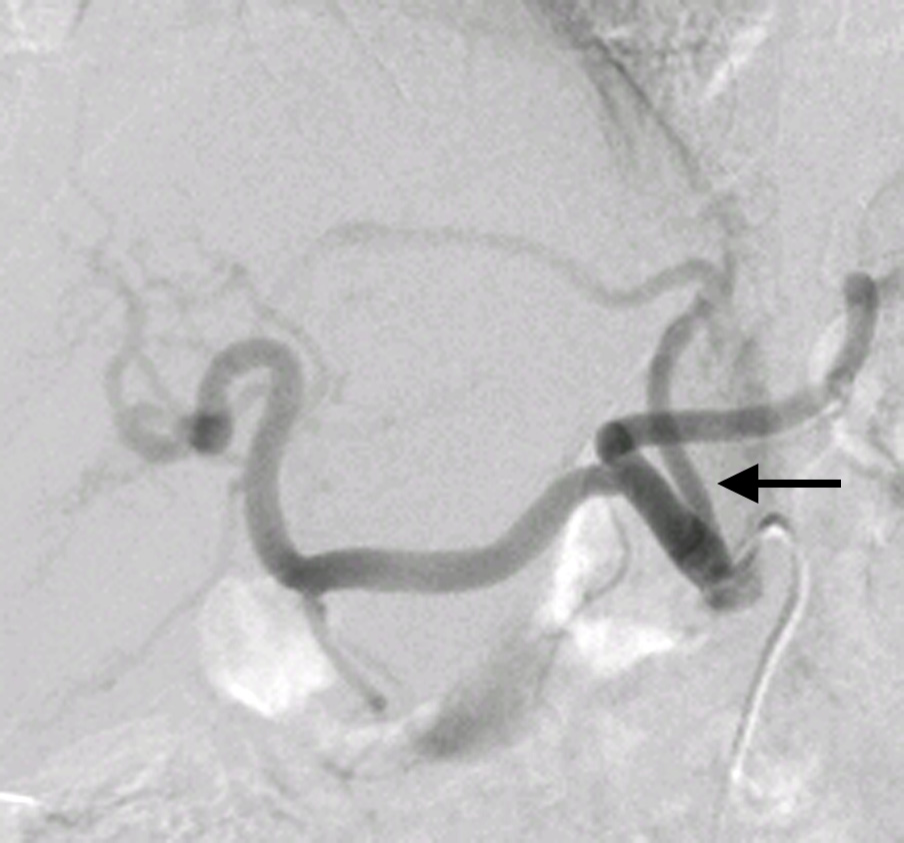
**Celiac angiogram (left anterior oblique, 30° angle) showing the left gastric artery (arrow) arising from the proximal portion of the up-swinging celiac trunk at a sharp angle**.

**Figure 3:**
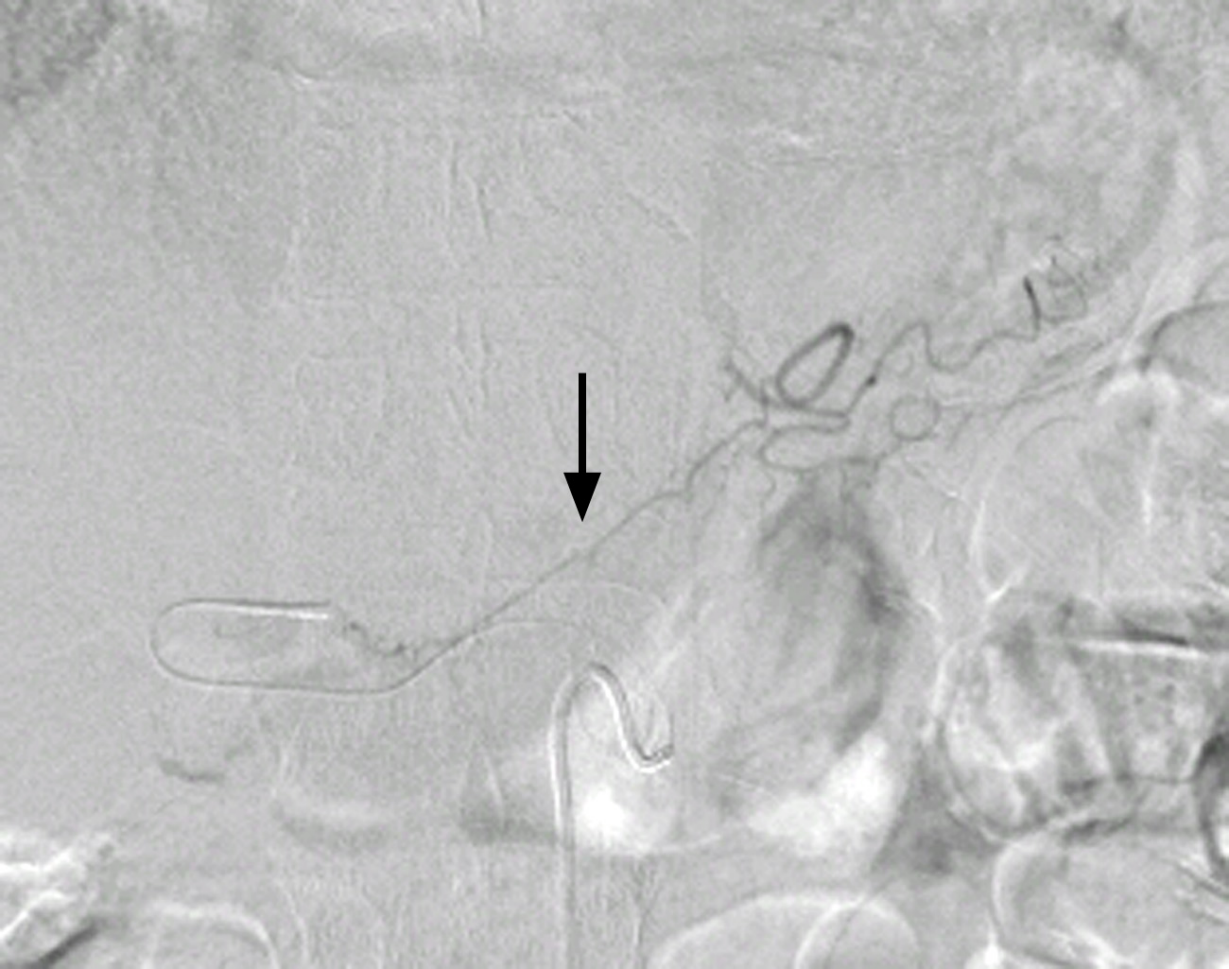
**Arteriogram obtained through the microcatheter inserted into the right gastric artery showing the very thin anastomosis (arrow) from the right gastric artery to the left gastric artery**. The replaced left hepatic artery cannot be seen through the anastomosis.

**Figure 4:**
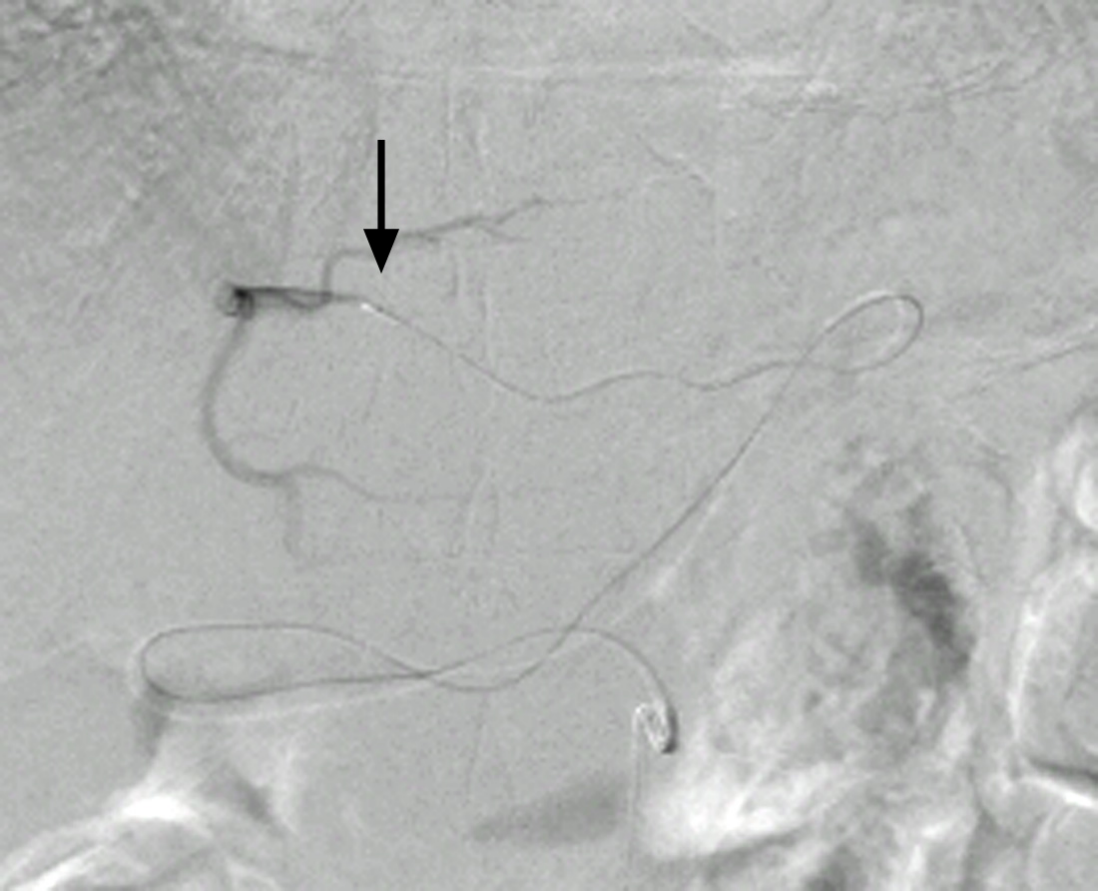
**The microcatheter (arrow) was successfully inserted into the distal portion of the replaced left hepatic artery via the right gastric artery through the anastomosis**.

**Figure 5:**
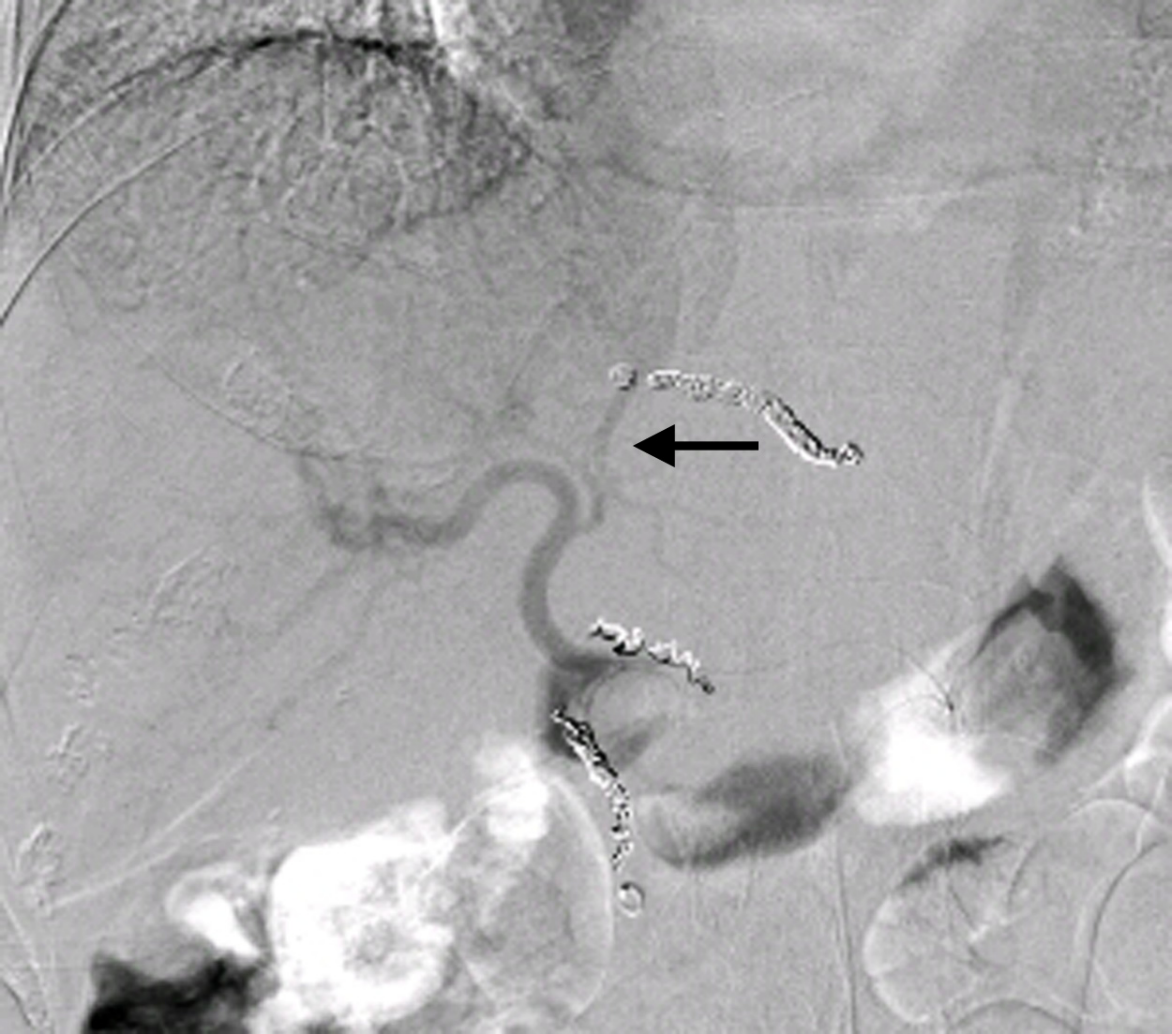
**Angiogram obtained through the implantable port after catheter placement showing the revascularized left hepatic artery (arrow) and a uniform blood supply to the entire liver**.

First, conventional angiography from the right femoral artery was performed so that we could visualize the anatomy. According to the celiac angiography, the LGA arose from the proximal portion of the up-swinging celiac trunk at a sharp angle, and no vascular stenosis was observed in the LGA (Figure 2). We attempted the following methods to catheterize the LGA: (1) turning the catheter tip to the up-swinging celiac trunk by pulling the 5-French shepherd's hook catheter (Terumo Clinical Supply, Tokyo, Japan) and trying to select the LGA by using a coaxial method with 2.1-French or 2.5-French microcatheters with or without the steam-shaped technique (Renegade, Boston Scientific, Natick, MA, USA, and Sniper 2, Terumo Clinical Supply) and 0.014-inch or 0.016-inch microguidewires (Transcend, Boston Scientific, GT wire, Terumo Clinical Supply); (2) inserting the steam-shaped 5-French shepherd's hook or Cobra catheter (Terumo Clinical Supply) into the common hepatic artery beyond the region of origin of the LGA and pulling them back to select the LGA; and (3) creating a side hole in the top of the shepherd's hook catheter and trying to insert the microcatheter into the LGA from the side hole. However, we failed to catheterize the LGA after trying all three methods, and the procedure time had reached about three hours. Therefore, using the microcatheter, we selected the RGA that arose from the proper hepatic artery. According to the RGA angiography, the anastomosis from the RGA to the LGA was very thin, and the replaced LHA was not visualized through the anastomosis at that time (Figure 3). However, we believed that the replaced LHA would be visualized if we inserted the catheter to the distal portion of the RGA and injected contrast medium into it. Therefore, we attempted to select the replaced LHA via the RGA and finally succeeded in visualizing and selecting it by using a 2-French microcatheter (Prograde-α; Terumo Clinical Supply) and 0.014-inch to 0.016-inch microguidewires (Transend, Boston Scientific, and GT wire, Terumo Clinical Supply) (Figure 4). The replaced LHA was embolized from the distal to the proximal portion using 13 microcoils (Tornado; Cook, Bloomington, IN, USA). The RGA was also embolized with three microcoils using the pull-back microcatheter. A 5-French polyurethane catheter with a 2.7-French distal shaft (W-Spiral catheter; Piolax Medical Devices, Yokohama, Japan) with a side hole was placed into the hepatic artery from the left femoral artery. The side hole was positioned in the common hepatic artery, and the tip was inserted into the gastroduodenal artery. After placing coils around the catheter tip to fix it within the gastroduodenal artery, the catheter was connected to an implantable port (Sadica port; Terumo Clinical Supply), which was embedded subcutaneously in the left anterior thigh. An angiogram obtained through the implantable port after catheter placement showed the revascularized LHA and a uniform blood supply to the entire liver (Figure 5). The total procedure time was four and a half hours. On the day after the procedure, hepatic arterial infusion chemotherapy was started and the patient was transferred to the previous hospital.

## Discussion

Repeat hepatic arterial infusion chemotherapy using an implanted port-catheter system is an accepted treatment for patients with unresectable advanced liver malignancies [5-7]. Recent advancements in interventional radiologic techniques have made insertion of the port-catheter system much easier [3,4].

Conversion of multiple hepatic arteries into a single vascular supply is a very important technique to use in this treatment. For patients with multiple hepatic arteries, all except the one to be used for chemotherapy infusion must be embolized so that drugs can be distributed to the entire liver using a single indwelling catheter [1,2,4].

A replaced right hepatic artery arising from a superior mesenteric artery and a replaced LHA arising from an LGA are the most common hepatic artery variants [1]. When a replaced LHA arising from an LGA is present, the proximal portion of the replaced LHA should be embolized with embolic materials. However, catheterizing an LGA using a percutaneous catheter technique is sometimes difficult, despite recent advanced interventional techniques. In most cases, an LGA can be catheterized easily using only a simple technique (for example, by turning the catheter tip to an up-swinging position by pulling the catheter). However, complicated techniques (for example, using the steam-shaped catheter or the catheter with a side hole) are occasionally needed to catheterize an LGA. In our patient, the causes of difficulties for catheterizing the LGA were assumed to be that (1) the LGA arose from the proximal portion of the up-swinging celiac trunk at a sharp angle, (2) vascular flexibility was lost because of arterial sclerosis, and (3) an undetectable intimal flap was present after multiple interventional treatments.

As is commonly known, the RGA generally anastomoses with the LGA. Some studies have reported the efficacy of catheter insertion for the RGA via the LGA through the anastomosis when catheterizing the RGA was difficult, and the RGA is then embolized to prevent a gastric ulcer during hepatic arterial infusion chemotherapy [8-10]. Alternatively, to the best of our knowledge, there have been no reports of catheterizing and embolizing the replaced LHA via the RGA through the anastomosis. In the present case, we inserted the catheter through the very thin anastomosis by using the appropriate microcatheters and microguidewires.

## Conclusion

Our case indicates that a replaced LHA arising from an LGA can be catheterized via the RGA through the anastomosis and that multiple hepatic arteries can be converted into a single supply by using our method, even if, despite the recent development of advanced interventional techniques, catheterizing the LGA is very difficult.

## Consent

Written informed consent was obtained from the patient for publication of this case report and any accompanying images. A copy of the written consent is available for review by the Editor-in-Chief of this journal.

## Competing interests

The authors declare that they have no competing interests.

## Authors' contributions

MM was involved in the conception of the report, the literature review, and manuscript preparation, editing, and submission. KS was involved in the clinical care of the patient. YT and KE were involved in manuscript editing and review. MM will act as guarantor for the manuscript. All authors read and approved the final manuscript.
